# Skillful statistical models to predict seasonal wind speed and solar radiation in a Yangtze River estuary case study

**DOI:** 10.1038/s41598-020-65281-w

**Published:** 2020-05-25

**Authors:** Peng Zeng, Xun Sun, David J. Farnham

**Affiliations:** 1 Key Laboratory of Geographic Information Science (Ministry of Education), East China Normal University, Shanghai, 200241 China; 2 School of Geographic Sciences, East China Normal University, Shanghai, 200241 China; 3 Department of Global Ecology, Carnegie Institution for Science, Stanford, CA USA

**Keywords:** Solar energy, Wind energy

## Abstract

This paper illustrates the potential for seasonal prediction of wind and solar energy resources through a case study in the Yangtze River estuary. Sea surface temperature and geopotential height-based climate predictors, each with high correlation to ensuing seasonal wind speed and solar radiation at the Baoshan weather observing station, are identified and used to build statistical models to predict seasonal wind speed and solar radiation. Leave-one-out-cross-validation is applied to verify the predictive skill of the best performing candidate model for each season. We find that predictive skill is highest for both wind speed and solar radiation during winter, and lowest during summer. Specifically, we find the most skill when using climate information from the July-September season to predict wind speed or solar radiation during the subsequent November-January season. The ability to predict wind and solar energy availability in the upcoming season can help energy system planners and operators anticipate seasonal surpluses or shortfalls and take precautionary actions.

## Introduction

Renewable energy resources, such as wind and solar power, play a crucial role in reducing the use of fossil fuels and ultimately lowering carbon dioxide emissions, mitigating anthropogenic global warming and enhancing drought resilience^1,2^. The desire to integrate renewable energy sources into energy systems exists in many regions of the world. One challenge that remains, however, is the substantial variability of wind and solar power availability^3–5^. As a consequence, accurately predicting variations in the availability of wind and solar resources is essential for energy system planning and operation.

A number of methods and models for forecasting wind and solar energy resources have been proposed. These methods can be mainly divided into two categories: statistical methods and physical models. Statistical models utilize historical time series data to estimate a statistical relationship between relevant explanatory variables and wind and solar energy availability. Regression and time series models, such as autoregressive (AR), moving average (MA) and autoregressive integrated moving average models (ARIMA)^6–9^, are often utilized. Physical models attempt to simulate the underlying physics associated with wind and solar energy, often within a numerical weather prediction (NWP) framework^10–12^. The efficacy of statistical and physical models depends on the desired prediction time horizon^13–17^. Consequently, a combination physical-statistical model has the potential to incorporate the strengths of each type of model and improve skill in predicting the variability of wind and solar energy resources at different time horizons.

There have been several prior studies regarding the influence of climate teleconnections on wind and solar energy resources. For instance, Ravestein, *et al*.^18^ discussed the impact of both climate change and climate variability on the supply of renewable energy sources in Europe. Berg, *et al*.^19^ and Mohammadi and Goudarzi^20^ investigated the sensitivity of wind speed and solar radiation in California to the El Nino Southern Oscillation (ENSO). Chen, *et al*.^21^ and Sherman, *et al*.^22^ summarized the decreasing potential and interannual variability of wind power in China. They showed that the information of the Pacific Decadal Oscillation (PDO), the Arctic Oscillation (AO) and the ENSO could be exploited to improve energy system management. Guo, *et al*.^23^ found that a weakening lower-tropospheric pressure-gradient between the land and sea in coastal China has been a primary cause of the observed decreasing trend in near-surface wind speed in that region. Most prior studies focus on characterizing the relationship between teleconnection indices and renewable energy variables and do not evaluate the efficacy of a model that uses climate information to predict wind and solar energy resources.

The goal of this paper is to present a predictive modeling framework for wind and solar energy resources at the seasonal timescale. In the remainder of the paper, we illustrate climate predictor identification, model selection, and prediction performance assessment for the case study region of the Yangtze River estuary. The Yangtze River estuary is the largest economic zone in China and rich in renewable energy resources^24–26^. A skillful prediction of seasonal wind and solar resources for this region can help facilitate the management and operation of the electricity system and can ultimately aid in the effort to integrate more wind and solar energy sources into the power system.

## Data Description

### Wind speed and solar radiation data

**Figure 1:**
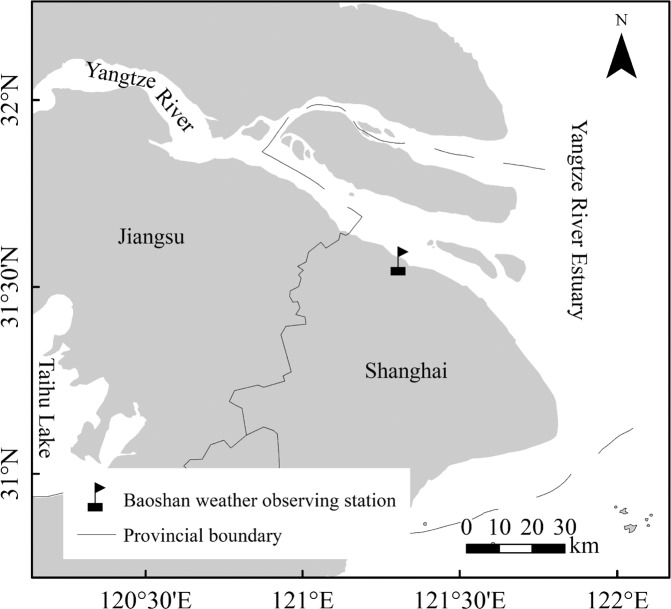
Location of the Baoshan weather observing station in the Yangtze River estuary.

Daily wind speed data from 1959 to 2017 and solar radiation data from 1958 to 2016 at Baoshan weather observing station (121.45°E, 31.4°N, Fig. 1) in the Yangtze River estuary were selected for this study. The wind speed was measured at 10 m above the ground. The data were provided by the National Climate Center, China Meteorological Administration. A three-month moving average of wind speed and solar radiation was calculated using the corresponding daily data. A preliminary analysis of the seasonality of solar radiation shows that the weakest and the strongest solar radiation occurs in the November-December-January (NDJ) and May-June-July-August (MJJA) seasons, respectively. Consequently, we define the four seasons as February-March-April (FMA), May-June-July (MJJ), August-September-October (ASO) and November-December-January (NDJ).

A trend analysis of the seasonal wind speed and solar radiation was conducted using a modified Mann-Kendall test^27^ that accounts for the reduced effective sample size resulting from serial correlation in the data. The results show that the seasonal wind speed for all four seasons and seasonal solar radiation for the MJJ and NDJ seasons have significant decreasing trends with *p*-values less than 0.05. Seasonal solar radiation during the FMA and ASO seasons do not exhibit statistically significant monotonic trends.

The modest negative trends in solar radiation may be the result of accelerated industrialization and burning of coal, which leads to increased near-surface aerosols^28^. In turn, these near-surface aerosols can lead to reduced incoming solar radiation at the surface due to a) their scattering and absorbing properties, and b) their promotion of reflectivity by clouds since aerosols can act as cloud condensation nuclei^29^.

Explaining the pronounced negative trends in wind speed is more complex because the mechanisms differ by season. The pronounced negative trends have been shown to be widespread throughout China in the historical record^23,30,31^ and a discussion of the possible mechanisms is included in Guo, *et al*.^23^. As mentioned above, a trend in the land-sea pressure gradient is one such proposed mechanism.

### Climate teleconnection and predictor identification

Several studies have reported strong teleconnections between precipitation in the study area and sea surface temperature (SST) anomalies in the Pacific and north Indian Oceans^32–34^. We can infer therefore that SST anomalies in particular ocean regions may affect cloud coverage in our study area. In addition, geopotential height (a large-scale climate field index related to surface pressure) is also related to wind patterns and cloud coverage. Therefore, to identify climate variables with the potential to predict season-ahead wind speed and solar radiation, we consider a) SST fields obtained from the Hadley Center SST dataset on a 1° × 1° grid^35^ and b) geopotential height at 850-hPa (GPH850) obtained from the National Center for Environmental Prediction–National Center for Atmospheric Research reanalysis data on a 2.5° × 2.5° grid^36^. We investigate the correlation between 3-month moving averaged SST and GPH850 with linearly de-trended wind speed and solar radiation for each season. For each season, we evaluate several correlations where the SST and GPH850 fields are leading the seasonal wind speed and solar radiation at the study site by between 6 and 0 months. Tables 1 and 2 summarize the regions with high correlation for all four seasons, in which the SST regions and GPH850 regions are separately listed. The selected regions of SST and GPH850 have significant and persistent correlations (i.e. the correlations persist when you evaluate concurrent and lagging correlations) with seasonal wind speeds and solar radiation at the study site. For example, a significant correlation between FMA wind speed and the SSTs in the region defined by 10°N~20°N and 135°E~235°E is identified not only for the concurrent season (i.e. wind speed of FMA and average SST of FMA), but also for prior season SSTs (i.e. wind speed of FMA and average SST of NDJ, OND, SON, ASO, etc.). Hence, using SST and GPH850 information can provide a lead time for predicting seasonal wind speed and solar radiation. We use 0.3 as a correlation threshold with which to identify potential predictor regions. This threshold of 0.3 was used to obtain a compromise between having sufficient lead time and obtaining skillful predictions.

**Table 1:** Summary of regions and time lags where the absolute correlation between seasonal wind speed and SST/GPH850 is larger than 0.3 with *p*-values less than 0.05.

Climate predictors	Season			
	FMA	MJJ	ASO	NDJ
SST1	10°N~20°N, 135°E~235°E[−5,0] negative correlation		13°N~23°N, 210°E~235°E[−6, 0]negative correlation	25°N~32°N, 145°E~160°E[−6,−2]positive correlation
SST2				37°N~47°N, 185°E~210°E[−6,−2]positive correlation
GPH850.1	0°N~25°N, 50°E~110°E[−6,−2] negative correlation		0°N~15°N, 110°E~160°E[−4,−1] negative correlation	−5°N~15°N, 60°E~115°E[−6,0] negative correlation
GPH850.2				35°N~50°N, 70°E~120°E[−6,−1] negative correlation

1 and 2 in SST and GPH850 are the index.

The temporal lags between the study site wind speed and SST/GPH850 are presented in the square brackets. Negative values indicate the months in which SST/GPH850 is ahead of the study site wind speed time series. The blank cells indicate that no SST/GPH850 region with spatially coherent and temporally persistent correlation to seasonal study site wind speed was identified.

**Table 2:** Summary of regions and time lags where the absolute correlation between seasonal solar radiation and SST/GPH850 is larger than 0.3 with *p*-values less than 0.05.

Climate predictors	Season			
	FMA	MJJ	ASO	NDJ
SST1	35°N~45°N, 175°E~200°E[−6,−1] positive correlation	40°N~45°N, 185°E~200°E[−6,0] positive correlation	40°N~50°N, 175°E~205°E[−6,0] positive correlation	10°N~25°N, 125°E~140°E[−5,0] positive correlation
SST2				−5°N~5°N, 180°E~240°E[−5, 0] negative correlation
GPH850.1	60°N~70°N, 100°E~140°E[−3,−1] positive correlation			−10°N~10°N, 60°E~85°E[−4, 0] negative correlation
GPH850.2	0°N~25°N, 50°E~100°E[−6, 0] negative correlation			10°N~30°N, 140°E~200°E[−6,−3] positive correlation

1 and 2 in SST and GPH850 are the index.

The temporal lags between the study site solar radiation and SST/GPH850 are presented in the square brackets. Negative values indicate the months in which SST/GPH850 is ahead of the study site solar radiation time series. The blank cells indicate that no SST/GPH850 region with spatially coherent and temporally persistent correlation to seasonal study site solar radiation was identified.

**Figure 2:**
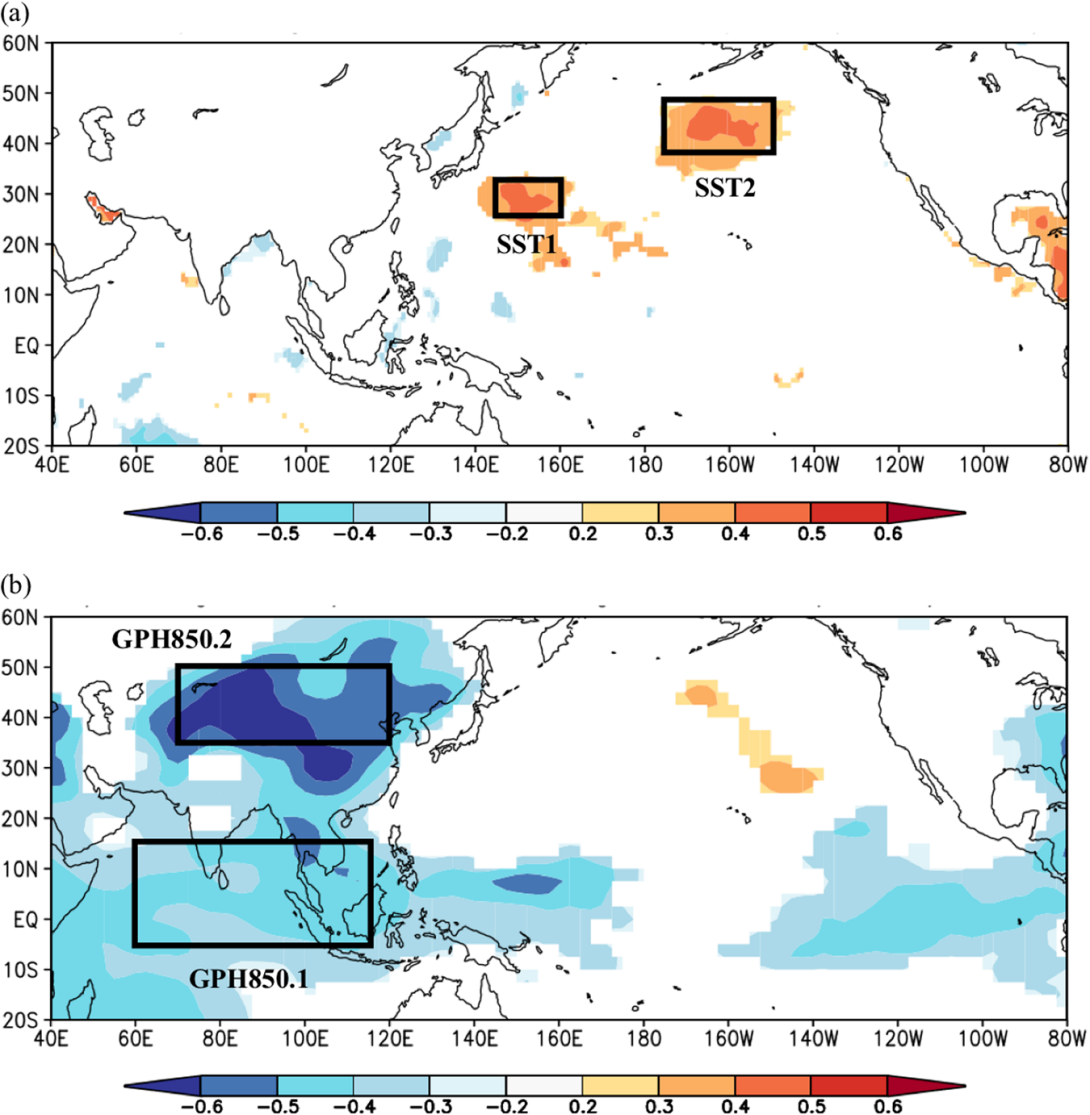
(**a**) Pearson correlation between the observed study site NDJ wind speed and the JAS SST. (**b**) Pearson correlation between the observed study site NDJ wind speed and the JAS GPH850. The regions with blue or red color show significant correlation at a level of 5%. The black boxes in (**a**) and (**b**) show where spatially coherent and temporally persistent correlations were found. The coordinates of the climate predictor regions are the following. SST1: 25°N-32°N, 145°E-160°E; SST2: 37°N-47°N, 185°E-150°W; GPH850.1: 5°S-15°N, 60°E-115°E; GPH850.2: 35°N-50°N, 70°E-120°E.

**Figure 3:**
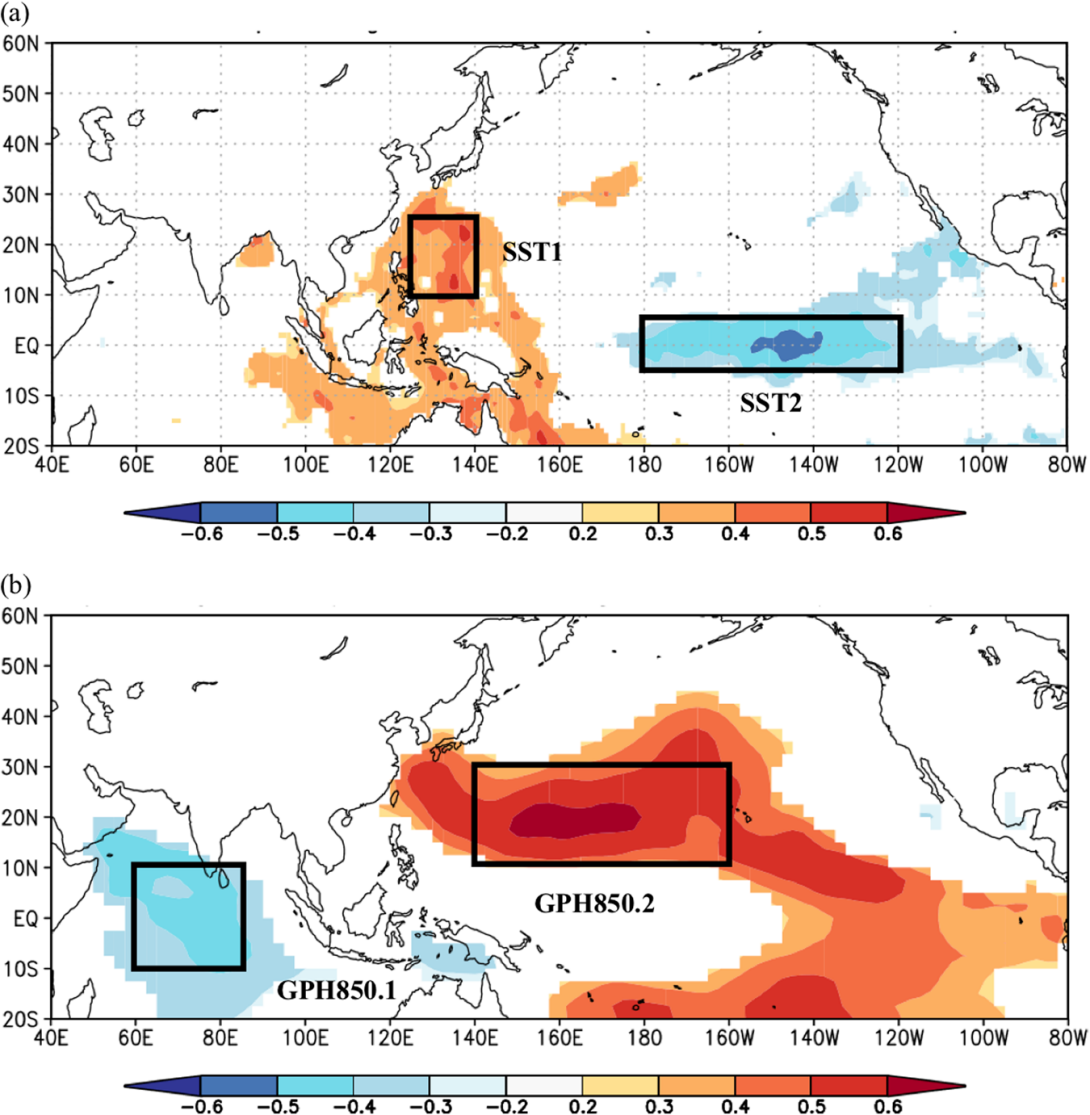
(**a**) Pearson correlation between the observed study site NDJ solar radiation and the JAS SST. (**b**) Pearson correlation between the observed study site NDJ solar radiation and the JAS GPH850. The regions with blue or red color show significant correlation at a level of 5%. The black boxes in (**a**) and (**b**) show where spatially coherent and temporally persistent correlations were found. The coordinates of the climate predictor regions are the following. SST1: 10°N-25°N, 125°E-140°E; SST2: −5°S-5°N, 180°-120°W; GPH850.1: 10°S-10°N, 60°E-85°E; GPH850.2: 10°N-30°N, 140°E-160°W.

Figures 2a,b and 3a,b show the Pearson correlation between the observed wind speed/solar radiation of NDJ and SST/GPH850 of JAS, respectively. Two regions with high correlation were identified in each figure. The SST/GPH850 within the identified regions may provide predictive information regarding the following season’s wind speed and solar radiation. A one-month lag between the climate information and predictand indicates that the wind speed/solar radiation of NDJ may be predictable using data that is available in the beginning of October. The SST and GPH850 regions that are correlated with wind speed and solar radiation at the study site over several months are summarized in Tables 1 and 2, respectively. To extract the climate predictors from the identified SST and GPH850 regions, Empirical Orthogonal Functions (EOFs) are used to extract the leading principle components (PCs) from the corresponding SST and GPH850 regions. We evaluated all components that explained more than 5% of variation but found that only PC1 of the SST/GPH fields in the regions of interest were useful. In each case PC1 explains more than 70% of the SST/GPH850 regional variance and has a correlation with the area-averaged SST/GPH850 with absolute value greater than 0.8. In other words, the information contained in PC1 is very similar to the information contained in the area-averaged SST/GPH850. We found, however, that using the leading component (rather than the area-averaged value) led to more skillful models.

We rely on cross-validation to limit the risk that our selected climate predictors are spuriously related to wind speed and solar radiation at our study site. As such, we do not focus on identifying the full causal pathway by which the identified climate predictors relate to our station-based wind speed and solar radiation. Having said that, we offer speculation on plausible physical connections between the predictors identified in Figs. 2 and 3 and wind speed and solar radiation at our study site in the Supplemental Information.

We also consider some well-known large-scale low-frequency climate variables such as SST ENSO indices (including Nino 4, Nino 3.4, Nino 3), the Pacific Decadal Oscillation (PDO), the North Atlantic Oscillation (NAO), the Southern Oscillation Index (SOI), and the Arctic Oscillation Index (AO) as candidate predictors of seasonal wind speed and solar radiation. For collinear indices, such as Nino 4, Nino 3.4, Nino 3, the SOI, and the PDO, only the index with the highest correlation to the wind and solar variables is used in the next step. Tables 3 and 4 summarize the selected predictors and their correlation with the study site wind speed and solar radiation time series for different seasons. These predictors contain climate information that may inform predictand variability at multiple timescales from interannual to interdecadal.

**Table 3:** The correlation between study site wind speed and climate predictors.

Climate predictors	Season			
	FMA	MJJ	ASO	NDJ
SST1_PC1	−0.36**(NDJ)		−0.45***(FMA)	0.45***(JAS)
SST2_PC1				0.41**(JAS)
GPH850.1_PC1	−0.45***(OND)		−0.44***(MJJ)	−0.45***(JAS)
GPH850.2_PC1				−0.60***(JAS)

() is the period of selected climate predictors; *indicating significance at the 0.05 level, and **indicating significance at the 0.01 level, and ***indicating significance at the 0.001 level.

**Table 4:** The correlation between study site solar radiation and climate predictors.

Climate predictors	Season			
	FMA	MJJ	ASO	NDJ
SST1_ PC1	0.49 *** (SON)	0.35 ** (DJF)	0.43 *** (AMJ)	0.49 ***(JAS)
SST2_ PC1				−0.47 ***(JAS)
GPH850.1_ PC1	0.44 *** (NDJ)			−0.42 *** (JAS)
GPH850.2_ PC1	−0.59 *** (NDJ)			0.62 *** (JAS)
SOI	0.35 ** (OND)			0.47*** (JAS)
NINO3.4				−0.48***(ASO)

() is the period of selected climate predictors; *indicating significance at the 0.05 level, and **indicating significance at the 0.01 level, and ***indicating significance at the 0.001 level.

## Methodology

### Linear temporal regression models to predict wind speed and solar radiation

Given the significant correlations of wind speed and solar radiation at the study site with the selected climate predictors as shown in Tables 3 and 4, we consider three linear temporal regression (LTR) models. Each season is studied independently.

Case 1: time-varying model (LTR_T_), which considers time as its sole predictor;

Case 2: univariate time-varying climate-informed regression model (LTR_U_), which considers one climate variable and time as its two predictors;

Case 3: multi-variable time-varying climate-informed regression model (LTR_M_), which considers several climate variables and time as predictors. The three linear temporal regression models are formulated as in Eq. (1).

1 $$LTR\,:W/\,S \sim {a}_{1}+{a}_{2}t+\mathop{\sum }\limits_{i}^{I}{b}_{i}{P}_{i}$$

In Eq. (1), *W/S* is wind speed or solar radiation, *t* is time, *P*_*i*_ is the climate variable, *I* is the total number of available climate predictors, and *a*_1_*, a*_2_ and *b*_*i*_ (*i* = 0, …, *I*) are the parameters to be estimated through a regression method. *I* = 0 corresponds to the LTR_T_ model, *I* = 1 corresponds to the LTR_U_ model, and *I* > = 2 corresponds to the LTR_M_ model.

### Model selection

The adjusted coefficient of determination (Adj.R^2^) is used to assess the goodness-of-fit of each model. The Akaike Information Criterion (AIC)^37^ is also used to compare different models and to select the best combination of climate variable predictors. The AIC is a goodness-of-fit estimator that accounts for model complexity in an effort to avoid overfitting (more precisely the AIC is two times the negative log likelihood plus two times the number of predictors in the model). Models with smaller AIC values and larger Adj.R^2^ values are preferred.

### Evaluation of prediction performance

Leave-one-out-cross-validation (LOOCV) is applied to estimate the predictive efficacy of the selected models. The procedure of LOOCV is the following. 1) set aside one year of data from the observational record to be used as testing data, 2) fit the model on the remaining (n-1) years of data, 3) predict the left-out year based on the model fit and the left-out year’s climate predictor values. This process is repeated for every year. Then, we select the optimal model among all candidate models by calculating the mean squared error (MSE) values under LOOCV and under full sample estimation, respectively. The mean squared error (MSE) is defined in Eq. (2).

2 $$MSE=\frac{1}{n}\mathop{\sum }\limits_{t=1}^{n}({O}_{t}-{P}_{t}){}^{2}$$

In Eq. (2), *O*_*t*_ and *P*_*t*_ are the observed and predicted wind speed or solar radiation values for year *t* and *n* is the number of years over which the MSE is computed.

## Results

### Goodness-of-fit and model selection

The AIC and Adj.R^2^ values for the seasonal wind speed and solar radiation candidate models are summarized in Tables 5 and 6. Models that include climate predictors perform better than those that only include temporal trends according to the AIC and Adj.R^2^ values. When multiple noncollinear climate predictors are available, models with multiple climate predictors show the best performance. This suggests that the information carried by the climate predictors improves the explanation of variance in seasonal wind speed and solar radiation at the study site.

**Table 5:** Fitting and prediction performance results for seasonal wind speed models.

Wind speed season	Predictors	AIC	Adj.R^2^	MSE
FMA	*t* (Case 1)	48	0.80	0.13
	*t*; SST1_PC1 (Case 2)	42	0.82	0.12
	*t*; GPH850.1-PC1 (Case 2) **	36	0.84	0.10
	*t*; SST1_PC1; GPH850.1_PC1 (Case 3)	38	0.83	0.11
MJJ	*t* (Case 1) **	36	0.78	0.10
ASO	*t* (Case 1)	61	0.62	0.16
	*t*; GPH850.1_PC1 (Case 2)	50	0.68	0.13
	*t*; SST1_PC1 (Case 2)	49	0.69	0.13
	*t*; SST1_PC1; GPH850.1_PC1 (Case 3) **	44	0.72	0.12
NDJ	*t* (Case 1)	49	0.80	0.13
	*t*; SST2_PC1 (Case 2)	40	0.83	0.11
	*t*; GPH850.1_PC1 (Case 2)	38	0.84	0.11
	*t*; SST1_PC1 (Case 2)	37	0.84	0.11
	*t*; GPH850.2_PC1 (Case 2)	24	0.87	0.09
	*t*; SST1_PC1; GPH850.1_PC1 (Case 3)	30	0.86	0.10
	*t*; SST1_PC1; GPH850.2_PC1 (Case 3) **	21	0.88	0.08

‘**’ indicates the best model for wind speed according to AIC and Adj.R^2^.

**Table 6:** Fitting and prediction performance results for seasonal solar radiation models.

Solar radiation season	Predictors	AIC	Adj.R^2^	MSE
FMA	*t* (Case 1)	479	−0.01	192.05
	SOI (Case 2)	472	0.11	167.59
	GPH850.1_PC1 (Case 2)	467	0.18	155.43
	SST1_PC1 (Case 2)	463	0.24	144.76
	GPH850.2-PC1 (Case 2)	455	0.33	142.75
	SST1-PC1; GPH850.1-PC1 (Case 3)	459	0.29	136.56
	GPH850.1-PC1; GPH850.2-PC1 (Case 3)	454	0.35	136.45
	SST1-PC1; GPH850.2_PC1 (Case 3) **	452	0.38	125.06
MJJ	*t* (Case 1)	502	0.11	280.32
	*t*; SST1-PC1 (Case 2) **	496	0.21	256.27
ASO	*t* (Case 1)	496	0.03	254.81
	SST1-PC1 (Case 2) **	487	0.17	222.67
NDJ	*t* (Case 1)	441	0.19	99.90
	*t*; GPH850.1-PC1 (Case 2)	431	0.32	85.25
	*t*; SST2_PC1 (Case 2)	428	0.36	79.27
	*t*; NINO3.4 (Case 2)	428	0.36	78.68
	*t*; SOI (Case 2)	427	0.37	79.54
	*t*; SST1-PC1 (Case 2)	426	0.38	78.17
	*t*; GPH850.2_PC1 (Case 2)	413	0.50	62.70
	*t*; GPH850.2_PC1; SOI (Case 3)	412	0.52	61.59
	*t*; GPH850.2-PC1; NINO3.4 (Case 3)	411	0.52	61.06
	*t*; GPH850.1_PC1; GPH850.2_PC1 (Case 3)	410	0.53	63.79
	*t*; SST1-PC1; GPH850.2-PC1 (Case 3)	410	0.53	59.10
	*t*; SST1_PC1; GPH850.1_PC1; GPH850.2_PC1 (Case 3) **	406	0.57	56.62

‘**’ indicates the best model for solar radiation according to AIC and Adj.R^2^.

### Effects of climate on seasonal wind and solar resources

**Figure 4:**
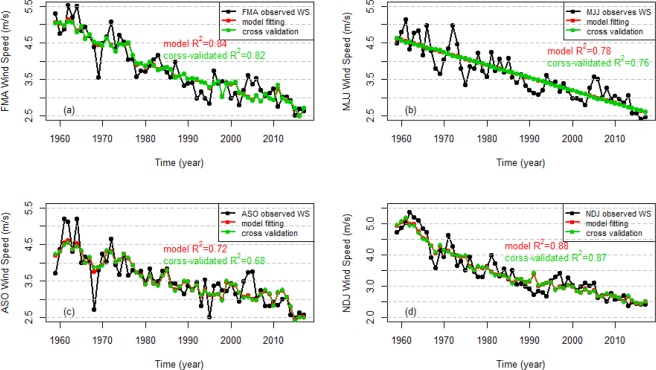
Time series of FMA (**a**), MJJ (**b**), ASO (**c**) and NDJ (**d**) observed wind speed (black solid circles and lines), model fits (red solid circles and lines) using the best models, and leave-one-out-cross-validated predictions (green solid circles and lines). The associated R^2^ values are shown in the corresponding colours.

In order to analyze the effects of the climate predictors on the seasonal wind speed and solar radiation variation, the Adj.R^2^ values for the models that only include temporal trend terms or climate predictor terms are shown in Table 7. For all models of wind speed, the temporal trend explains the majority of variance, specifically between 62% and 80% depending on the season. The climate predictors explain 19%, 26%, and 40% of the remaining variance during FMA, ASO, NDJ, respectively. This indicates that although the negative temporal trend (illustrated in Fig. 4a–d) explains the majority of interannual wind speed variance, the climate predictors also explain a substantial portion of wind speed variation for three of the seasons. For solar radiation, no significant trends were identified in FMA and ASO, while weak decreasing trends were found in MJJ and NDJ. These trends account for 11% and 19% of the variance in the MJJ and NDJ seasons, respectively. Climate predictors explain 38%, 11%, 17%, 47% of the remaining interannual solar radiation variance during FMA, MJJ, ASO, NDJ, respectively. The primary takeaway is that the inclusion of climate predictors in the seasonal wind speed and solar radiation models generally leads to substantial increases in predictand variance being explained (Table 7).

**Table 7:** Summary of the fitting performance of the best models for wind speed and solar radiation only using the linear temporal trend or climate variables as predictors.

Season	Temporal trend Adj.R^2^	Climate variable Adj.R^2^
Feb-Apr wind speed	0.80	0.19
May-Jul wind speed	0.78	
Aug-Oct wind speed	0.62	0.26
Nov-Jan wind speed	0.80	0.40
Feb-Apr solar radiation		0.38
May-Jul solar radiation	0.11	0.11
Aug-Oct solar radiation		0.17
Nov-Jan solar radiation	0.19	0.47

The blank cells indicate that the best model for that season and variable did not include a temporal trend term (middle column) or climate predictor terms (right column). The Adj.R^2^ values for the models that only include climate predictor terms are calculated using the data after removing the temporal trend of observed wind speed and solar radiation.

### Validation of the prediction models

**Figure 5:**
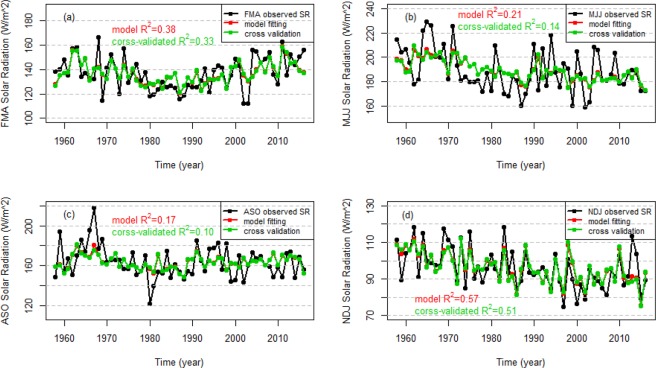
Time series of FMA (**a**), MJJ (**b**), ASO (**c**) and NDJ (**d**) observed solar radiation (black solid circles and lines), model fits (red solid circles and lines) using the best models selected, and leave-one-out-cross-validated predictions (green solid circles and lines). The associated R^2^ values are shown in the corresponding colours.

LOOCV is applied to assess the predictive skill of the seasonal wind speed and solar radiation models and the MSE values for all the models are shown in Tables 5 and 6. The models that include climate predictors generally have smaller MSE values compared to the models that do not include climate predictors. This increased skill under cross-validation provides evidence that the use of climate information increases the predictive capacity of the models. Figures 4a–d and 5a–d show the time series of observed seasonal wind speed and solar radiation, model fits, and the cross-validated predictions for the models with the lowest MSE for each season. The model fits and cross-validation predictions are generally very similar. This indicates a low risk of overfitting. For both wind speed and solar radiation, the FMA and NDJ models have higher cross-validated R^2^ values than the MJJ and ASO models. The NDJ season model has the best cross-validated R^2^ of all solar radiation models, which is in part because the linear trend is most pronounced in that season.

**Figure 6:**
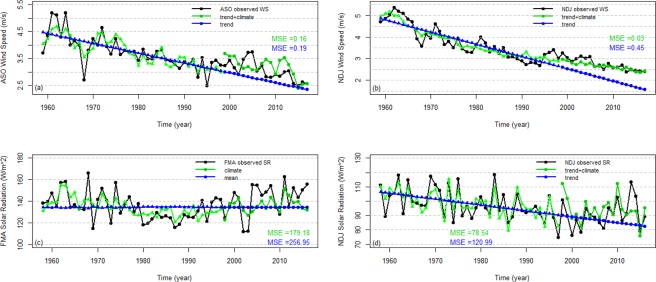
Comparison between the observed ASO, NDJ wind speed (**a,b**), FMA, NDJ solar radiation (**c,d**) (black solid circles and lines) and model fits and predictions based on the selected models for the calibration (1959–1998 for wind speed, 1958–1997 for solar radiation) and validation (1999–2017 for wind speed, 1998–2016 for solar radiation) periods (green and blue circles and lines). Green circles and lines represent the models incorporating climate predictors, and blue circles and lines represent either the time series mean or models that only include the linear temporal trend term.

In addition to cross-validation, we also illustrate the predictive performance of the models when the data are separated into calibration and validation periods. The calibration and validation periods are 1959–1998 and 1999–2017 for wind speed and 1958–1997 and 1998–2016 for solar radiation. Figure 6a–d show the comparison between the observed and predicted ASO, NDJ wind speed, and FMA, NDJ solar radiation. Consistent with the LOOCV results, the models that include climate predictors outperform the models that do not include climate predictors on the basis of MSE over the validation period. It is notable that the climate predictors appear to capture changes in the low-frequency signals that occur between the calibration and validation periods and that are not captured by the linear trend term (see ASO and NDJ wind speeds in Fig. 6). While the climate predictors do improve the model performance during the validation period, the predictions still deviate substantially from the observations in some respects. For example, in general the variance of the predictions is smaller than the variance of the observations (e.g. FMA solar radiation), and some large observed anomalies are not explained by our models (e.g. see years 2008–2009 for ASO wind speed and years 2002–2005 for FMA solar radiation in Fig. 6). It should also be noted that the climate predictor identification phase implicitly utilized information from both the LOOCV left-out years and from the validation period since the predictors were selected based on the full dataset. However, our method yields the same sets of predictors whether we use the full dataset or the training period data to select model predictors.

## Summary and Conclusions

The main objective of this study was to develop a skillful model based on climate information to predict seasonal wind speed and solar radiation in the Yangtze River estuary. First, we identified SST regions, GPH850 regions, and some standard climate indices that have a strong correlation with ensuing seasonal wind speed and solar radiation at the Baoshan observing station. Second, we developed predictive models based on the identified climate predictors. Three linear regression models were considered in this study: a time-varying model, a univariate time-varying climate-informed model, and a multi-variable time-varying climate-informed model. The AIC and Adj.R^2^ were applied to select the best-fitting model for each of the four seasons. Third, we conducted a cross-validation analysis on the selected models to check that the models were not overfitted and to evaluate how much of the interannual wind speed and solar radiation variability could be predicted by the models. The results demonstrate that both the newly derived large-scale SST and GPH850 indices as well as pre-existing climate indices can explain a portion of the impact of large-scale climate circulations on the variability of local wind and solar energy availability. The models presented in this paper illustrate the ability to skillfully predict wind speed or solar radiation at the seasonal timescale.

Seasonal prediction of wind speed and solar radiation has the potential to help facilitate integration of wind and solar electricity generation into existing electricity grids by allowing grid operators to better plan for potential surpluses or shortfalls in upcoming seasonal generation. For example, knowing in advance that there is increased risk of low wind power generation for an upcoming season could be useful for several entities. This information could 1) allow turbine owners to schedule maintenance during these seasons so as to limit the costs associated with such maintenance, or 2) allow energy managers to better anticipate the need for back-up fuels, such as natural gas, during the upcoming season.

Efforts to investigate the predictability of wind and solar resources are becoming increasingly salient as many regions worldwide increase their dependence on these renewable resources. As such, further research into the predictability of wind and solar power availability at timescales from sub-daily to decadal is warranted. While the climate variables that best predict seasonal wind speed and solar radiation will vary from region to region, the modeling framework of this study can be expanded to other regions in China as well as other countries.

## Supplementary information

Supplementary Information.

## References

[1] Edenhofer, O. *et al*. IPCC, 2011: IPCC special report on renewable energy sources and climate change mitigation, Working Group III of the Intergovernmental Panel on Climate Change. (2011).

[2] He X (2019). Solar and wind energy enhances drought resilience and groundwater sustainability. Nat Commun.

[3] Beluco A, Souza PKD, Krenzinger A (2008). A dimensionless index evaluating the time complementarity between solar and hydraulic energies. Renewable Energy.

[4] Coker P, Barlow J, Cockerill T, Shipworth D (2013). Measuring significant variability characteristics: An assessment of three UK renewables. Renewable Energy.

[5] François B (2016). Complementarity between solar and hydro power: Sensitivity study to climate characteristics in Northern-Italy. Renewable Energy.

[6] Chen P, Pedersen T, Bak-Jensen B, Zhe C (2010). ARIMA-Based Time Series Model of Stochastic Wind Power Generation. IEEE Transactions on Power Systems.

[7] Firat, U., Engin, S. N., Saraclar, M. & Ertuzun, A. B. in Ninth International Conference on Machine Learning & Applications.

[8] Mora-López L, Sidrach-De-Cardona M (1998). Multiplicative ARMA models to generate hourly series of global irradiation. Solar Energy.

[9] Shi J, Qu X, Zeng S (2011). Short-Term Wind Power Generation Forecasting: Direct Versus Indirect Arima-Based Approaches. International Journal of Green Energy.

[10] Foley AM, Leahy PG, Marvuglia A, Mckeogh EJ (2012). Current methods and advances in forecasting of wind power generation. Renewable Energy.

[11] Cornaro C (2015). Twenty-Four Hour Solar Irradiance Forecast Based on Neural Networks and Numerical Weather Prediction. Journal of Solar Energy Engineering.

[12] Johnson DL, Erhardt RJ (2016). Projected impacts of climate change on wind energy density in the United States. Renewable Energy.

[13] Aggarwal SK (2013). Wind Power Forecasting: A Review of Statistical Models. International. Journal of Energy Science.

[14] Diagne M, David M, Lauret P, Boland J, Schmutz N (2013). Review of solar irradiance forecasting methods and a proposition for small-scale insular grids. Renewable & Sustainable Energy Reviews.

[15] Inman RH, Pedro HTC, Coimbra CFM (2013). Solar forecasting methods for renewable energy integration. Progress in Energy & Combustion Science.

[16] Ssekulima E, Anwar MB, Hinai AA, Elmorusi MS (2016). Wind Speed and Solar Irradiance Forecasting Techniques for Enhanced Renewable Energy Integration with the Grid; A Review. Iet Renewable Power Generation.

[17] Wang X, Peng G, Huang X (2011). A Review of Wind Power Forecasting Models. Energy Procedia.

[18] Ravestein P (2018). Vulnerability of European intermittent renewable energy supply to climate change and climate variability. Renewable and Sustainable Energy Reviews.

[19] Berg N, Hall A, Capps SB, Hughes M (2013). El Niño-Southern Oscillation impacts on winter winds over Southern California. Climate Dynamics.

[20] Mohammadi K, Goudarzi N (2018). Study of inter-correlations of solar radiation, wind speed and precipitation under the influence of El Niño Southern Oscillation (ENSO) in California. Renewable Energy.

[21] Chen L, Li D, Pryor SC (2013). Wind speed trends over China: quantifying the magnitude and assessing causality. International Journal of Climatology.

[22] Sherman P, Chen X, Mcelroy MB (2017). Wind-generated Electricity in China: Decreasing Potential, Inter-annual Variability and Association with Changing Climate. Scientific Reports.

[23] Guo H, Xu M, Hu Q (2011). Changes in near‐surface wind speed in China: 1969–2005. International Journal of Climatology.

[24] Dong S, Gong Y, Wang Z, Incecik A (2019). Wind and wave energy resources assessment around the Yangtze River Delta. Ocean Engineering.

[25] Gosens, J., Kåberger, T. & Wang, Y. China’s next renewable energy revolution: goals and mechanisms in the 13th Five Year Plan for energy. Energy Science & Engineering 5 (2017).

[26] Hong L, Xie M, Zhang T (2013). Promote the development of renewable energy: A review and empirical study of wind power in China. Renewable & Sustainable Energy Reviews.

[27] Yue S, Wang C (2004). The Mann-Kendall Test Modified by Effective Sample Size to Detect Trend in Serially Correlated Hydrological Series. Water Resources Management.

[28] Zhang, Y. L., Qin, B. Q. & Chen, W. M. Analysis of 40 year records of solar radiation data in Shanghai, Nanjing and Hangzhou in Eastern China. Theoretical & Applied Climatology 78, 217-227.

[29] Wild & Martin. Decadal changes in radiative fluxes at land and ocean surfaces and their relevance for global warming. Wiley Interdisciplinary Reviews Climate Change 7, 91–107.

[30] Jiang, Y., Luo, Y., Zhao, Z. & Tao, S. Changes in wind speed over China during 1956–2004. Theoretical & Applied Climatology 99, 421-430.

[31] Lin, C., Yang, K., Qin, J. & Fu, R. Observed Coherent Trends of Surface and Upper-Air Wind Speed over China since 1960. Journal of Climate 26, 2891–2903.

[32] Chang CP, Zhang Y, Li T (2000). Interannual and Interdecadal Variations of the East Asian Summer Monsoon and Tropical Pacific SSTs. Part II: Meridional Structure of the Monsoon. Journal of Climate.

[33] Hyunhan K, Brown C, Xu KQ, Lall U (2009). Seasonal and annual maximum streamflow forecasting using climate information: application to the Three Gorges Dam in the Yangtze River basin, China. International Association of Scientific Hydrology Bulletin.

[34] Yang F, Lau KM (2004). Trend and variability of China precipitation in spring and summer: linkage to sea‐surface temperatures. International Journal of Climatology.

[35] Rayner, N. A. *et al*. Global analyses of sea surface temperature, sea ice, and night marine air temperature since the late nineteenth century. Journal of Geophysical Research 108, (2003).

[36] Kalnay, E. *et al*. The NCEPNCAR 40-Year Reanalysis Project. (1996).

[37] Akaike H (1974). A new look at the statistical model identification. IEEE Transactions on Automatic Control.

