# Supplementary material for: Skillful statistical models to predict seasonal wind speed and solar radiation in a Yangtze River estuary case study
Source: Sci Rep. 2020 May 25;10:8597. doi: 10.1038/s41598-020-65281-w (PMC7248103; doi:10.1038/s41598-020-65281-w)
Supplement: Supplementary file 1 — Supplementary Information. [file 41598_2020_65281_MOESM1_ESM.pdf]

Supplementary Information for Skillful statistical models to predict seasonal wind speed and solar radiation in a Yangtze River estuary case study

Peng Zeng[1,2] , Xun Sun[1,2], David J. Farnham[3]

Correspondence

Xun Sun, Key Laboratory of Geographic Information Science (Ministry of Education), East China Normal University, Shanghai, 200241, China. E-mail: xs2226@columbia.edu

[1] Key Laboratory of Geographic Information Science (Ministry of Education), East China Normal University, Shanghai 200241, China.

[2] School of Geographic Sciences, East China Normal University, Shanghai 200241, China.

[3] Department of Global Ecology, Carnegie Institution for Science, Stanford, CA, USA.

Discussion of climate predictors shown in Figures 2 and 3

Below we present speculations regarding the associations between wind speed and solar radiation at our study site and the sea surface temperature (SST) and geopotential height at 850-hPa (GPH850) climate predictors that are identified in figures 2 and 3. We stress that these are speculations. Further diagnostic studies are needed in order to confidently understand causal pathways, or associations, between the identified climate predictors and wind speed and solar radiation at our study site.

The positive relationship between SST regions SST1 and SST2 in figure 2a and wind speed in the study region may be partially explained by the North Pacific jet stream. Specifically, elevated SSTs in these regions may be associated with a modulated North Pacific jet stream, which in turn could lead to modulated mean winds in the study region.

GPH850 anomalies in the GPH850.1 region of figure 2b appear to be associated with coastal wind anomalies near the study site (based on climate diagnostics). More specifically, negative GPH850 anomalies in the GPH850.1 region of figure 2b are associated with the La Niña phase of ENSO, which is in turn associated with enhanced winds in the coastal study region. The GPH850 in the GPH850.2 region of figure 2b appears to be associated with a modulated large-scale land-sea pressure gradient. Changes to this gradient can in turn influence coastal wind speeds including those at the study site.

The SSTs in the SST1 region of figure 3a may influence solar radiation at the study location by modulating on- and off-shore airflow. Modulated onshore winds may impact the aerosol concentration in the air column, and/or cloudiness, around the study site through the delivery of relatively clean ocean air. The SSTs in the SST2 region of figure 3a are closely related to a developing ENSO event. A La Niña event appears to be associated with onshore wind anomalies in the study region (based on climate diagnostics), which in turn could increase solar radiation at the study site through the delivery of relatively clean ocean air. La Niña is associated with negative SST anomalies in the central equatorial Pacific Ocean, thus explaining the negative sign of the correlation between the SST2 region of figure 3a and solar radiation at the study site.

Positive GPH850 anomalies in the GPH850.1 region of figure 3b appear to be associated with suppressed winds near the study region (based on climate diagnostics), which in turn may lead to more stagnant air, higher aerosol concentrations, and thus lower solar radiation at the surface. Positive GPH850 anomalies in the GPH850.2 region of figure 3b may be associated with increased surface solar radiation at the study site through modulated regional wind patterns that enhance onshore winds, which in turn deliver cleaner ocean air from the Pacific Ocean and East China Sea.
